# Resilience and perceived social support among family Caregivers of patients with mental illness : what links ?

**DOI:** 10.1192/j.eurpsy.2022.1602

**Published:** 2022-09-01

**Authors:** K. Safa, N. Charfi, M. Marwa, N. Smaoui, I. Gassara, R. Feki, S. Omri, M. Maalej, L. Zouari, J. Ben Thabet, M. Maalej

**Affiliations:** Hedi Chaker University hospital, Psychiatry C, sfax, Tunisia

**Keywords:** social support-resilience-caregivers-mental illness

## Abstract

**Introduction:**

Burden among family caregivers of patients with mental illness can be mediated by resilience and perceived social support.

**Objectives:**

The present study aimed to explore the relationship between perceived social support and resilience levels among caregivers of patients with mental illness and to identify its associated factors.

**Methods:**

This was a cross-sectional, descriptive and analytical study conducted on caregivers of patients followed in the outpatient psychiatry department at the university hospital of Sfax (Tunisia), during septembre 2021. Resilience was assessed with the Connor–Davidson Resilience Scale and the social support with the social provisions scale. High scores indicate high resilience and support.

**Results:**

We have involved 34 family caregivers with an average age of 47.47 years(SD=12.4 years). The mean resilience score of caregivers was 42.85 and the mean social support score was 28.09(SD=5.54). The score of resilience correlated positively with the score of social support among family caregivers of patients (r= 0.76 ; p=0.0001). Furthermore, resilience and social support were significantly lower among caregivers with a history of chronic illness (p=0.0001 and 0.002 respectively). Regarding clinical factors, the scores of social support and resilience decreased significantly with the duration of providing care to patients (r=-0.60; p=0.0001 and r=-0.75; p=0.0001 respectively). Similarly, they decreased significantly with the number of hospitalizations of patients (r=-0.53; p=0.001 and r=-0.62; p=0.0001 respectively).

**Conclusions:**

The findings suggests that high social support promotes better resilience among family caregivers of patients with mental illness. So, a better adjustement to the role of caregiver can be effectively achieved by improving the perceived social support.

**Disclosure:**

No significant relationships.

